# The first mitochondrial genome of *Capitulum mitella* (Crustacea: Cirripedia) from China: revealed the phylogenetic relationship within Thoracica

**DOI:** 10.1080/23802359.2020.1781564

**Published:** 2020-06-24

**Authors:** Mei Tian, Panpan Chen, Jun Song, Fuxiang He, Xin Shen

**Affiliations:** a Jiangsu Key Laboratory of Marine Biotechnology, Jiangsu Institute of Marine Resources, Jiangsu Ocean University, Lianyungang, China; b Co-Innovation Center of Jiangsu Marine Bio-industry Technology, Jiangsu Ocean University, Lianyungang, China; c Guangdong Provincial Key Laboratory of Marine Biotechnology, Shantou University, Shantou, China

**Keywords:** *Capitulum mitella*, mitochondrial genome, phylogeny, Crustacea, Cirripedia

## Abstract

*Capitulum mitella* (Crustacea: Cirripedia) is an important stalked barnacle. The first mitochondrial genome of *C*. *mitella* from China was presented, which is a circular molecule of 15,930 bp in length and AT content is 64.4%. It encodes 37 genes, including 13 PCGs, 22 tRNAs, and two rRNAs, which is consistent with most barnacles species reported. There are 15 genes encoded on the light strand and 22 genes encoded on the heavy strand. Identical to most barnacles species reported, *srRNA* and *lrRNA* genes are adjacent and separated only by *trnV* gene. Phylogenetic trees showed that *C*. *mitella* clustered with *Pollicipes polymerus*, indicating Pollicipedidae is monophyletic. However, Scalpelliformes was not monophyletic from the phylogenetic tree. From the level of order, the Lepadiformes was located at the base of the phylogenetic tree, indicating that its divergence time was earlier than Scalpelliformes. The results provided more insights into phylogenetic consideration at the genomic level within superorder Thoracica.

*Capitulum mitella* (Crustacea: Cirripedia), distributing on the rocks of intertidal zone, is an important stalked barnacle (Liu and Ren 2007), which has been studied for its ecological and economic value (Lee et al. 2000; Yoon et al.^2013^; Yuan et al. 2016). Recently, more and more species of Thoracica in Cirripedia and even deep-sea species have been obtained, constantly updating our understanding of the classification of superorder Thoracica (Chan et al. 2019). However, little is known about the phylogenetic relationship among the orders within Thoracica.

The specimen of *C. mitella* was collected from Zhoushan (30°43′1.64″N, 122°46′3.25″E), Zhejiang Province, China. The total DNA was extracted from muscle tissue, using TIANamp Marine Animal DNA Kit (TIANGEN), which was stored at Marine Museum of Jiangsu Ocean University (Accession number: Cmi-002). Sixty pairs of specific primers were designed with reference to the mitogenome of *Pollicipes mitella* (Lim and Hwang 2006). The DNA fragments were obtained by polymerase chain reaction amplification and sequenced by primer-walking strategy. MITOS (Bernt et al. 2013) and tRNAscan-SE (Chan and Lowe 2019) online website were used to finish the gene annotation.

The mitochondrial genome of *C. mitella* is 14,914 bp in length (GenBank accession number: MH119184; 64.4% AT content) and encoded a set of 37 typical metazoan mitochondrial genes, including 13 PCG, two rRNA, 22 tRNA genes, and one control region (Kim et al. 2017). There were 15 genes encoded on the light strand (including four PCGs and two rRNAs: *nad1*, *nad4*, *nad4L*, *nad5*, *srRNA*, and *lrRNA*), and the remaining 22 genes were transcribed on the heavy strand. The base composition of *C. mitella* is 33.85% A, 22.06% C, 12.62% G, and 31.42% T. AT and GC skews of the whole genome are −0.186 and −0.028, respectively.

In 13 PCGs, except that *cox1* and *nad1* genes started with ‘CGA’ and ‘TAT’ respectively, the remaining 11 genes started with ‘ATN’ (ATA, ATG or ATC). In addition, for stop codons, three genes (*cox3*, *nad3* and *nad4*) end with incomplete ‘T––’, the other 10 PCGs terminate with complete stop codons ‘TAA’ or ‘TAG’. The *srRNA* (752 bp; 64.0% AT content) and *lrRNA* (1351 bp; 68.7% AT content) of *C. mitella* were arranged continuously and separated only by the *trnV* gene, which is consistent with most barnacle species reported (Shen, Chan, et al. 2015; Shen, Tsang, et al. 2015; Shen et al. 2016).

Phylogenetic tree was constructed based on nucleotide data of 13 PCGs from 20 barnacles species and 2 polychaetes species (outgroup) with PhyloSuite software (Zhang et al. 2020) and MEGA 7.0.25 (Kumar et al. 2016), and iTOLs (Letunic and Bork 2016) website was used to draw the phylogenetic tree (Figure 1). *Capitulum mitella* clustered with *Pollicipes polymerus*, indicating Pollicipedidae is monophyletic. However, the three families under the same order Scalpelliformes were not clustered together, indicating this order is not monophyletic, which is consistent with Lee et al. (2019). Lepadiformes was located at the base of the phylogenetic tree, the divergence time was earlier than Scalpelliformes, which is similar to the result of nuclear gene analysis (Perez-Losada et al. 2008). Our results will contribute to the understanding of the phylogenetic history within superorder Thoracica.

**Figure 1.**
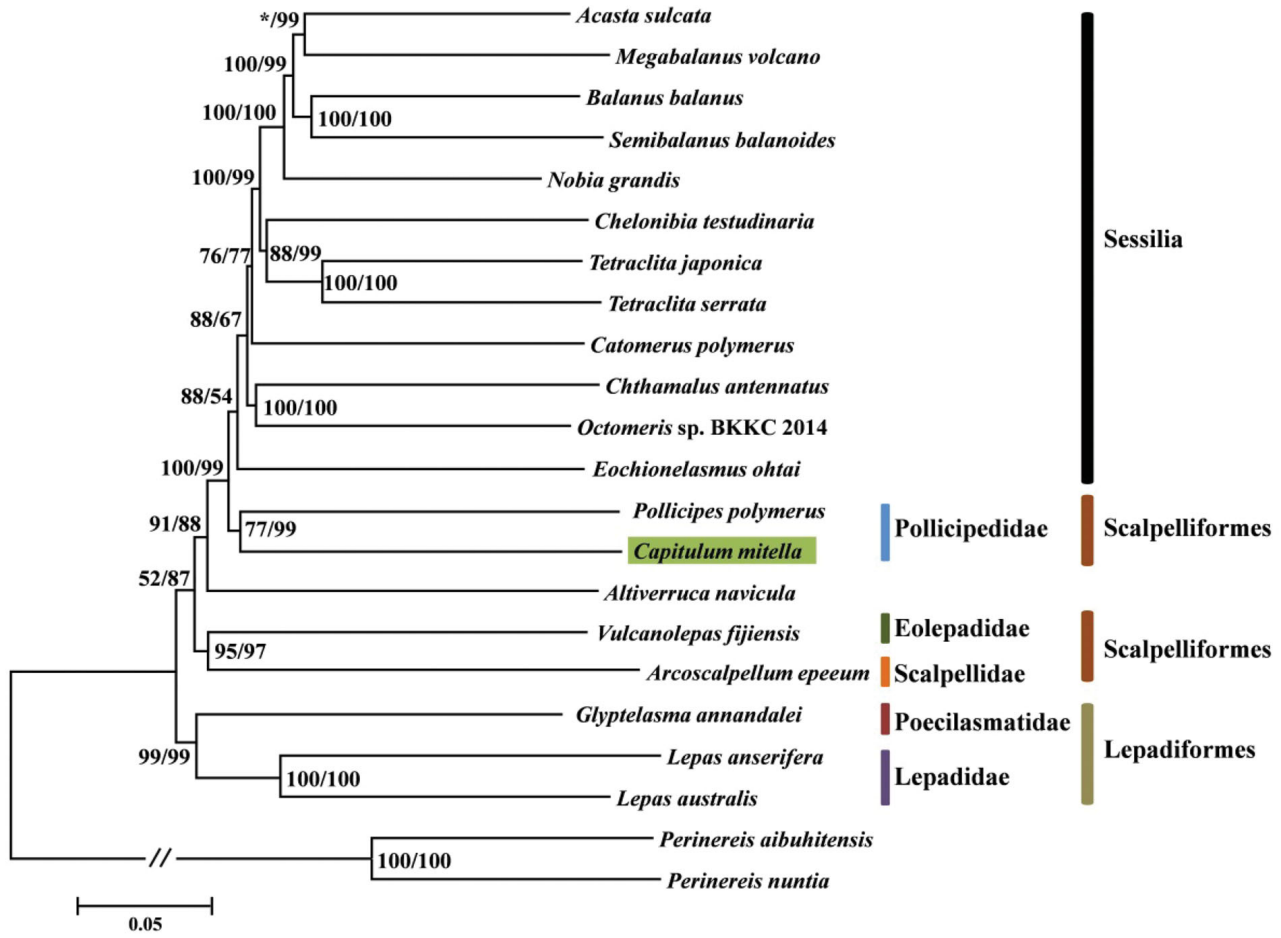
Phylogenetic trees of *Capitulum mitella* and other barnacles based on nucleotide data of 13 PCGs. Green-shaded box represents *C. mitella*. Different colors indicate different orders or families. The numerical values at the node represent the bootstrap value from MEGA (left) and PhyloSuite (right) software, respectively. ‘*’ represents bootstrap value is less than 50.

The accession numbers of the genomes used for comparison were NC_029168 (*Acasta sulcata*); NC_006293 (*Megabalanus volcano*); NC_026466 (*Balanus balanus*); NC_039849 (*Semibalanus balanoides*); NC_023945 (*Nobia grandis*); NC_029169 (*Chelonibia testudinaria*); NC_008974 (*Tetraclita japonica*); NC_029154 (*Tetraclita serrata*); MH791045 (*Catomerus polymerus*); NC_026730 (*Chthamalus antennatus*); KJ754820 (*Octomeris* sp. BKKC_2014); NC_036957 (*Eochionelasmus ohtai*); NC_005936 (*Pollicipes polymerus*); MH119184 (*Capitulum mitella*); NC_037244 (*Altiverruca navicula*); MN061491 (*Vulcanolepas fijiensis*); MH791047 (*Arcoscalpellum epeeum*); MH891848 (*Glyptelasma annandalei*); NC_026576 (*Lepas anserifera*); NC_025295 (*Lepas australis*); NC_023943 (*Perinereis aibuhitensis*); NC_020609 (*Perinereis nuntia*).

## Data Availability

The data that support the findings of this study are openly available in GenBank of NCBI at https://www.ncbi.nlm.nih.gov, reference number MH119184.
